# Behavioral response to heat stress of twig-nesting canopy ants

**DOI:** 10.1007/s00442-022-05143-6

**Published:** 2022-03-07

**Authors:** Jelena Bujan, Stephen P. Yanoviak

**Affiliations:** 1 Department of Biology, University of Louisville, Louisville, KY USA; 2 Department of Ecology and Evolution, University of Lausanne, 1015 Lausanne, Switzerland; 3 Smithsonian Tropical Research Institute, Balboa, Panama

**Keywords:** Formicidae, Heat stress, Insects, Ectotherms, Thermal tolerance

## Abstract

Tropical forests experience a relatively stable climate, but are not thermally uniform. The tropical forest canopy is hotter and thermally more variable than the understory. Heat stress in the canopy is expected to increase with global warming, potentially threatening its inhabitants. Here, we assess the impact of heating on the most abundant tropical canopy arthropods—ants. While foragers can escape hot branches, brood and workers inside twig nests might be unable to avoid heat stress. We examined nest choice and absconding behavior—nest evacuation in response to heat stress—of four common twig-nesting ant genera. We found that genera nesting almost exclusively in the canopy occupy smaller cavities compared to *Camponotus* and *Crematogaster* that nest across all forest strata. *Crematogaster* ants absconded at the lowest temperatures in heating experiments with both natural and artificial nests. *Cephalotes* workers were overall less likely to abscond from their nests. This is the first test of behavioral thermoregulation in tropical forest canopy ants, and it highlights different strategies and sensitivities to heat stress. Behavioral avoidance is the first line of defense against heat stress and will be crucial for small ectotherms facing increasing regional and local temperatures.

**Supplementary Information:**

The online version contains supplementary material available at 10.1007/s00442-022-05143-6.

## Introduction

Tropical forests are biodiversity hotspots characterized by a relatively stable climate (Janzen 1967; Ghalambor et al. 2006), but they are not thermally uniform. The tropical forest canopy is on average hotter and more variable than the litter below (Kumagai et al. 2001; Bujan et al. 2016). Surface temperatures of branches in lowland tropical forests can exceed 50 °C (Kaspari et al. 2015; Stark et al. 2017). These thermal extremes likely are challenging for ectotherms whose metabolism and fitness are governed by local environmental temperatures (Deutsch et al. 2008; Angilletta 2009). Ectotherms in the lowland tropics could be exposed to habitat temperature maxima that are close to or higher than their physiological tolerance, and must use behavioral avoidance to escape overheating (Sunday et al. 2014). Within a tropical forest, canopy ectotherms are already experiencing higher temperatures than their understory counterparts.

The most abundant tropical canopy arthropods are the ants (Davidson 1997). They comprise over 90% of total arthropod abundance in tropical tree crowns (Blüthgen and Stork 2007; Dejean et al. 2007). Canopy ants are expected to face even higher temperatures with ongoing global warming (Warren et al. 2018), deforestation, and habitat fragmentation (Wright 2005). Changes in microclimate caused by deforestation reduce the abundance of tropical ground-nesting ants, selecting for more thermally tolerant genera in disturbed, hotter habitats (Boyle et al. 2020). Such changes in abundance and community composition are concerning, because ants are ubiquitous and provide a variety of important ecosystem functions (Folgarait 1998; Philpott and Armbrecht 2006; Clay et al. 2013). Relative to ground-nesting ants, canopy ants commonly are exposed to potential heat stress and high thermal variability. We do not know how increased temperatures will affect canopy ants, or the importance of behavioral mechanisms used by arboreal ants to avoid thermal stress.

Unlike canopy ants, ground-nesting ants can escape unfavorable temperatures by altering nest chamber position and depth (Tschinkel 1987; Chick et al. 2017). Similarly, mound-nesting ants can cool down the nest by decreasing mound height and enlarging the openings (Horstmann and Schmid 1986; Jones and Oldroyd 2006). Most canopy ants nest in cavities of stems, trunks, and branches (Carroll 1979; Philpott and Foster 2005; Tanaka et al. 2010; Camarota et al. 2016) that cannot be modified quickly to facilitate thermoregulation. While foragers easily avoid extreme temperatures by changing foraging times or choosing to forage on cooler substrates (Spicer et al. 2017; Stark et al. 2017), queens, brood, and nurses inside the cavity nest are less likely to escape thermal extremes. Exposure to thermal extremes, or frequent temperature variation, can be detrimental to ant colonies, because proper brood development requires optimal temperature (Abril et al. 2010; Oms et al. 2017). Since twig-nesting ants cannot rapidly alter their nest properties, they must either withstand thermal stress or abscond (i.e., evacuate the nest).

Absconding often occurs in social insects that temporarily or permanently abandon their nest in response to unfavorable abiotic (e.g., temperature, flooding) or biotic conditions; e.g., predators, pathogens (Winston et al. 1979; Heinrich 1993). Ant colony absconding occurs in response to disturbance, such as army ant attacks, which causes workers to evacuate with brood (Droual 1983; Le Breton et al. 2007; Dejean et al. 2014). Litter nesting ants often abscond their nests to move to a superior nest site (Dornhaus et al. 2004). Thus, absconding behavior is a relatively common phenomenon among ants and occurs in response to a variety of stimuli. Given that leaf litter and other microhabitats in the forest understory experience high disturbance frequency (relative to canopy microhabitats) from physical factors and army ant raids (Kaspari 1996; Kaspari et al. 2011), we expect absconding behavior to be more common in ants that nest both in the canopy and the understory.

Here, we evaluated behavioral responses of twig-nesting tropical canopy ants to heat stress. We focused on four main questions: (1) How do the physical characteristics of twig nests shape their thermal properties and occupancy by focal ant genera?; (2) Are certain genera more likely to abscond in response to heat stress?; (3) Does thermal tolerance predict absconding temperature?; and (4) Does the presence of brood inside the nest promote absconding behavior? We experimentally heated natural and artificial twigs to quantify the behavioral responses of cavity-nesting ants to heat stress. We focused on ants of four common arboreal twig-nesting genera that differ in body size and life-history strategies.

## Materials and methods

We conducted this study on Barro Colorado Island (BCI; 9° 10′ N, 79° 51′ W) in Panama. BCI is a tropical lowland moist forest with an average monthly temperature of 27 °C and average annual rainfall of ca. 2600 mm (Leigh 1999). The BCI forest canopy is on average warmer than the understory (Bujan et al. 2016), with surface temperatures in tree crowns sometimes exceeding 50 °C on cloudless days (Kaspari et al. 2015; Stark et al. 2017).

We focused on species from four common canopy ant genera in our natural and artificial nest trials (Table S1). Two of these (*Cephalotes* and *Pseudomyrmex*) are almost exclusively canopy nesters, while the other two (*Camponotus* and *Crematogaster*) are found nesting in all forest strata, from the litter up to the canopy (Table S1). Thus, our four focal genera show differences in life-history strategies and span a broad range of body size both of which are likely relevant to their thermal tolerance limits (Table S2).

### Measuring nest characteristics

We collected cylindrical natural nests occupied by the four focal genera from the canopy and understory of the BCI forest. We used calipers to measure the diameter of the nest cavity and thickness of the wood on two opposite sides of the twig to the nearest 0.1 mm. Twig cavity shape is highly variable, so we measured overall nest diameter externally (Byrne 1994). We limited nest collections to twig nests that were around 1 cm in diameter (median ± SE = 0.88 ± 0.04), a single outlier was a 3 cm woody stem. We calculated the total nest volume and cavity volume using the formula for cylinder volume (*V* = π*r*^2^*l*). Where *r* is the radius calculated as half of the total diameter or cavity diameter, and *l* is the length of the nests used in the experiments. To calculate the volume of the woody part of the nest, we subtracted cavity volume from the total nest volume.

### Heating of natural nests

We trimmed all collected nests to 15 cm maximum length, so they could be evenly heated with a single 125 W UVA/UVB mercury vapor heat lamp (*d* = 18 cm; Exo-Terra Solar Glo; Rolf C. Hagen, Inc.; Mansfield, MA, USA) in the lab. We used Type K thermocouples (model TP-01; Reed Instruments, Wilmington, NC, USA) to measure air temperature inside the nest, at the exterior underside of the nest, and 5 cm away from the nest (i.e., ambient air). The thermocouples were secured in place using insulated garden wire. The ants were allowed to acclimate to the presence of the thermocouples overnight, during which the twig nest-thermocouple assembly was housed in a Fluon-lined plastic container at 24 °C. During this time, the ants were provided honey water ad libitum.

We transferred each nest with the thermocouple to a plastic container (32 × 19 × 12 cm) lined with Fluon applied to the top inner margin to prevent ant escape. Two insulated garden wires supported the nest 5 cm above the bottom of the container to prevent excessively rapid heating (Fig. S1). The three thermocouples described above were connected to a four-channel data logger (model RDXL4SD; Omega Engineering, Stamford, CT, USA). The heat lamp was positioned 23.5 cm away from the nest surface at the beginning of the experiment. When the recorded temperature was stable for 1 min, the experimental container was gradually elevated closer to the lamp to generate an average heating rate of 0.87 ± 0.04 °C min^−1^ (range = 0.4–2.1 °C min^−1^). We stopped the heating trial when the temperature inside the nest reached 50 °C, as this was near the maximum temperature previously recorded on branch surfaces in the canopy of this forest (Kaspari et al. 2015; Stark et al. 2017). We did not record the surface temperatures needed to attain an internal temperature of 50 ºC. However, we conducted a preliminary survey of nest temperatures in the tree crowns by inserting thermocouples in the middle of natural nests of different cavity volumes and recording temperature inside of the nest every 2 s, for 2–3 days. Maximum temperature recorded inside of natural nests of different sizes exceed 40 °C in almost ¼ of the nests (Table S3).

During the heating trials, we recorded the number of ants outside the nest at 1-min intervals. We also recorded the time and temperature of any observed changes in their behavior. We recorded the time and temperature at which the first worker exited the nest, and if and when any brood were carried out. We recorded *absconding temperature* as the temperature corresponding to a rapid mass exodus of workers from the nest (usually > 10 workers exiting fast, at the same time). Absconding was obvious for most genera, except *Pseudomyrmex* workers, which usually exited singly. Thus, for *Pseudomyrmex*, we recorded absconding temperature as the temperature at which the maximum number of workers was out of the nest. At the end of each heating trial, we sealed the nest entrances with cotton and counted the number of nest members found outside of the nest (i.e., workers, brood, males, virgin queens, and queens). We then opened the nest and counted the remaining nest members inside the twig. Most lab heating nest trials were done in the morning or afternoon, and we tested for the effect of time of day on absconding temperature and proportion of absconded workers.

### Heating of artificial nests

Natural nest architecture and properties (e.g., wood thickness, cavity size, wood density, initial moisture content) were highly variable, so we repeated the heating experiment with ants occupying artificial nests (cardboard cylinders 0.4 cm in diameter and 15 cm long). We evicted ants from their natural nests and offered them the cardboard nest with a thermocouple pre-installed in the middle. Artificial heating trials were conducted as described above with an average heating rate of 0.85 ± 0.02 °C min^−1^ (range = 0.5–1.3 °C min^−1^).

We chose cardboard nests, because they were readily accepted as surrogate nest sites by all four focal genera. Other materials like plastic straws and glass tubes were never accepted by *Cephalotes* spp. and were rejected by some species of *Pseudomyrmex*. We used glass test tubes covered with red cellophane paper to simulate a dark environment for large *Camponotus* species (e.g., *C. atriceps*), because these species were too large to occupy the cardboard nests. *Camponotus* ants readily colonized any provided cavity.

### Data analysis

We used Kruskal–Wallis tests to compare nest properties across ant genera, as the data were not normally distributed and sample sizes differed among groups. We used Dunn’s post hoc test with adjusted *P* values for multiple comparisons (Holm 1979) to determine significant differences among genera. We used generalized linear mixed-effect models (GLMMs) with a *glmer* function and binomial error distribution to determine which factors best predict the proportion of workers outside the nest at the end of a heating trial (i.e., absconded workers).

We examined the variability in absconding temperature in natural and artificial nests with the *lmer* function using a Gaussian error distribution (*lmerTest* package; Kuznetsova et al. 2017). In both cases, we used genus and proportion of brood in the nest as fixed factors. Nest identity was treated as a random factor. We used pairwise comparisons to determine differences between genera with the *emmeans* function (Lenth et al. 2018). To examine the effect of circadian rhythm on absconding temperature and the proportion of absconded workers, we added start hour as fixed factor to the full model. Time of day did not affect absconding temperature in natural (*F*_1,56_ = 0.12, *p* = 0.73) or artificial nests (*F*_1,58_ = 0.24, *p* = 0.62). The proportion of absconded workers was also not affected by the time of day in natural or artificial nests, so this variable was omitted from subsequent analyses (GLMM_natural_: *β* = 0.04, SE = 0.02, *z* (1.5), *p* = 0.12; GLMM_artificial_: *β* = 0.02, SE = 0.04, *z* (0.5), *p* = 0.63). All the analyses were performed in R version 3.6.2 (R Core Team 2019).

## Results

### Nest specificity

**Fig. 1:**
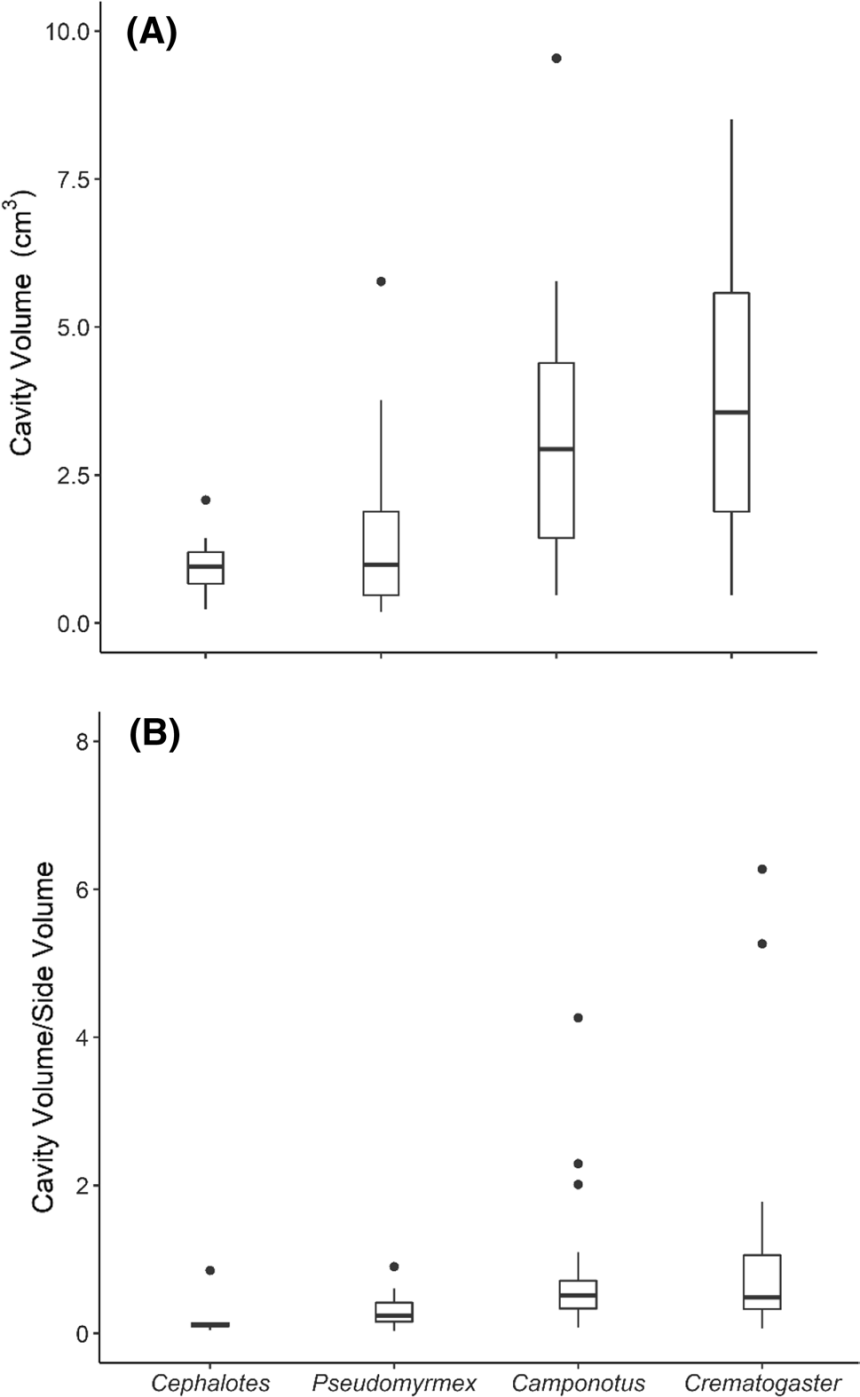
Box-and-whisker plots of cavity volume (**A**) and the ratio of cavity volume and side volume (**B**) of natural twig nests for the four focal genera used in this study: *Cephalotes* (*N* = 7), *Pseudomyrmex* (*N* = 23), *Camponotus* (*N* = 35)*, *and *Crematogaster* (*N* = 23). Sample size (N) refers to the total number of nests tested

**Fig. 2:**
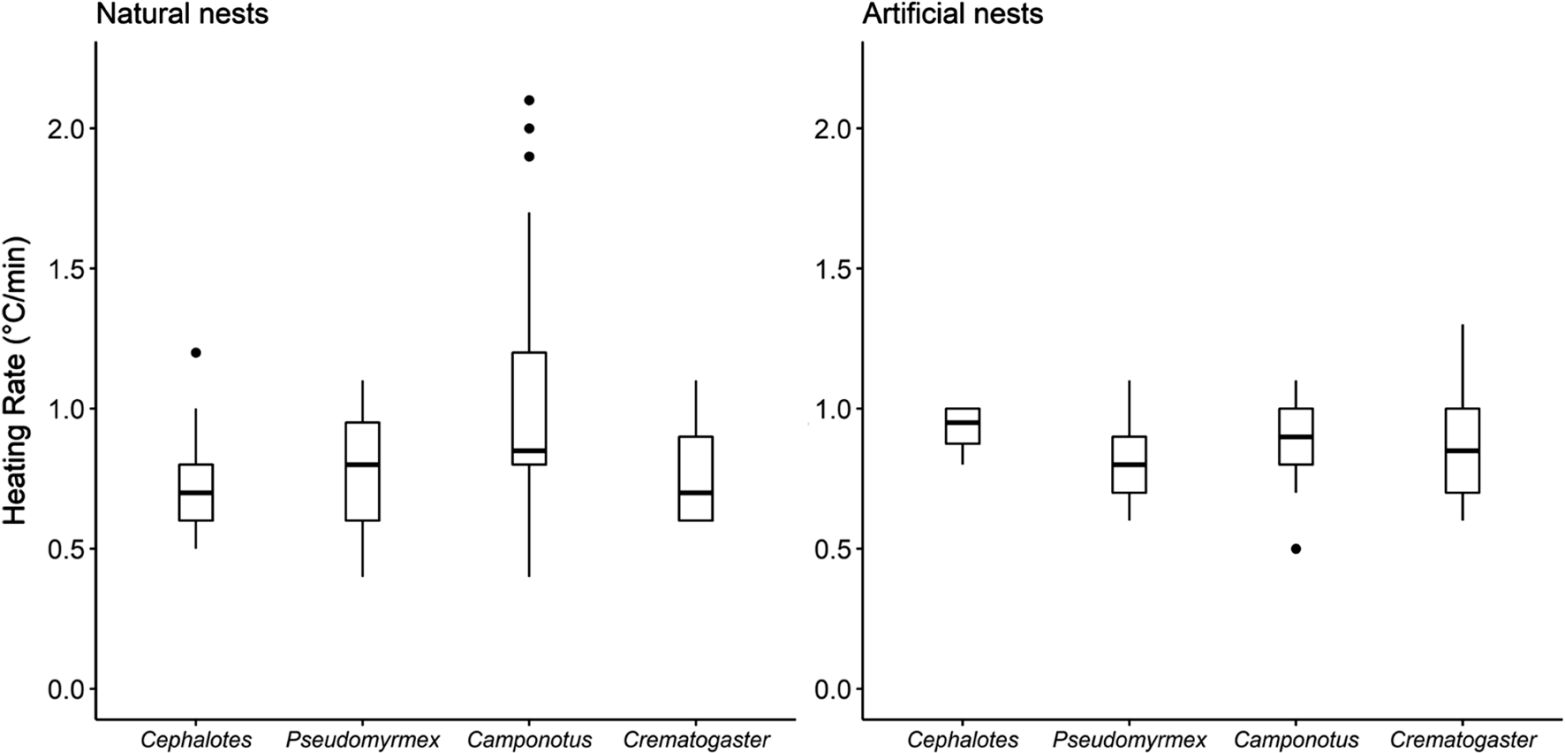
Heating rate of four focal genera measured in natural nests (left panel) and artificial nests (right panel). Only *Cephalotes* nests were slower to heat up compared to *Camponotus* in natural nests (*p* = 0.03), based on Dunn’s post hoc tests

The common canopy nesting genera, *Cephalotes* and *Pseudomyrmex*, collected in this study nested in smaller and less size-variable cavities than *Camponotus* and *Crematogaster* (Fig. 1A, *χ*^2^ = 25.2, df = 3, *p* < 0.001). The total volume of the wooded nest cylinder did not differ among focal ant genera (*χ*^2^ = 2.85, df = 3, *p* = 0.42). Nests of *Cephalotes* and *Pseudomyrmex* had a low cavity-to-wood ratio compared to the nests of *Camponotus* and *Crematogaster* (Fig. 1B; *χ*^2^ = 19.6, df = 3, *p* = 0.0002). Thus, genera that were predominantly canopy nesters (Table S1, *Cephalotes* and *Pseudomyrmex*) were found in twigs with relatively small cavities, and high proportion of wood relative to the cavity size. Their nests were usually tightly packed with workers and brood. *Cephalotes* nests were slower to heat up compared to the nests of *Camponotus* (Fig. 2; *χ*^2^ = 9.0, df = 3, *p* = 0.03). However, the heating rates of artificial nests did not differ between genera (Fig. 2; *χ*^2^ = 5.4, df = 3, *p* = 0.14).

### Absconding temperature

**Fig. 3:**
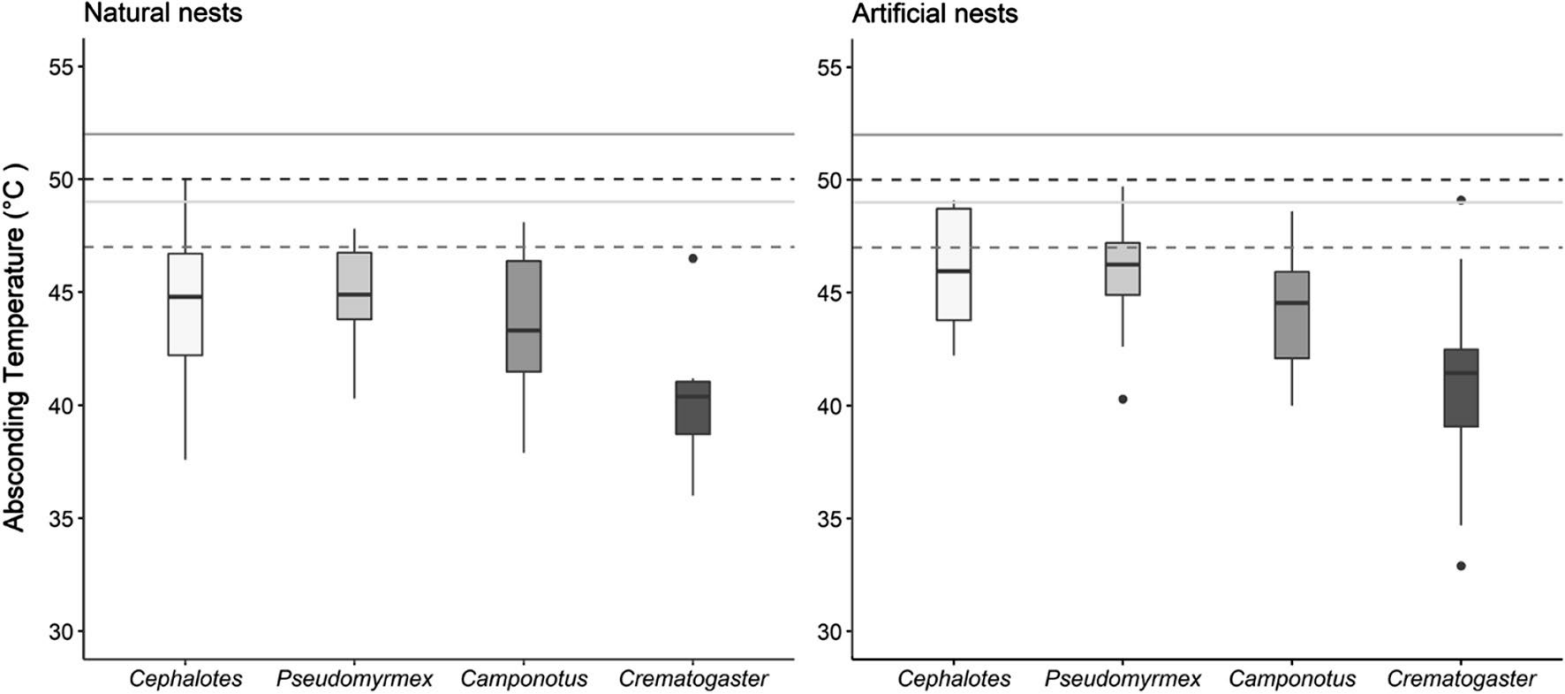
Absconding temperature across genera in natural and artificial nests. Only *Crematogaster* absconded at significantly lower temperatures compared to other genera in natural (*p* = 0.005) and artificial nests (*p* < 0.001). Horizontal lines represent average critical thermal maximum (CT_max_) for each genus. CT_max_ of *Cephalotes* and *Pseudomyrmex* are shown with solid lines in light gray color, and *Camponotus* and *Crematogaster* in dark gray and dashed lines. Colors of horizontal lines correspond to boxplot colors of each genus

Overall, *Crematogaster* absconded at a lower average temperature than all other genera in natural (40.2 ± 1.1 ºC) and artificial nests (41.0 ± 0.9 ºC). Genus identity was the only significant predictor of absconding temperature in both natural (Fig. 3; *F*_3,36_ = 5.0, *p* = 0.005) and artificial nests (Fig. 3; *F*_3,24_ = 8.8, *p* < 0.001). Although the optimal model contained the proportion of brood per nest, this variable was not a significant predictor of absconding temperature. Average absconding temperatures were generally higher in artificial vs. natural nests for all genera except *Camponotus,* which consistently absconded at 44 °C. *Cephalotes* and *Pseudomyrmex* absconded at the same temperature in both types of nests (Fig. 3).

### Absconding behavior

**Fig. 4:**
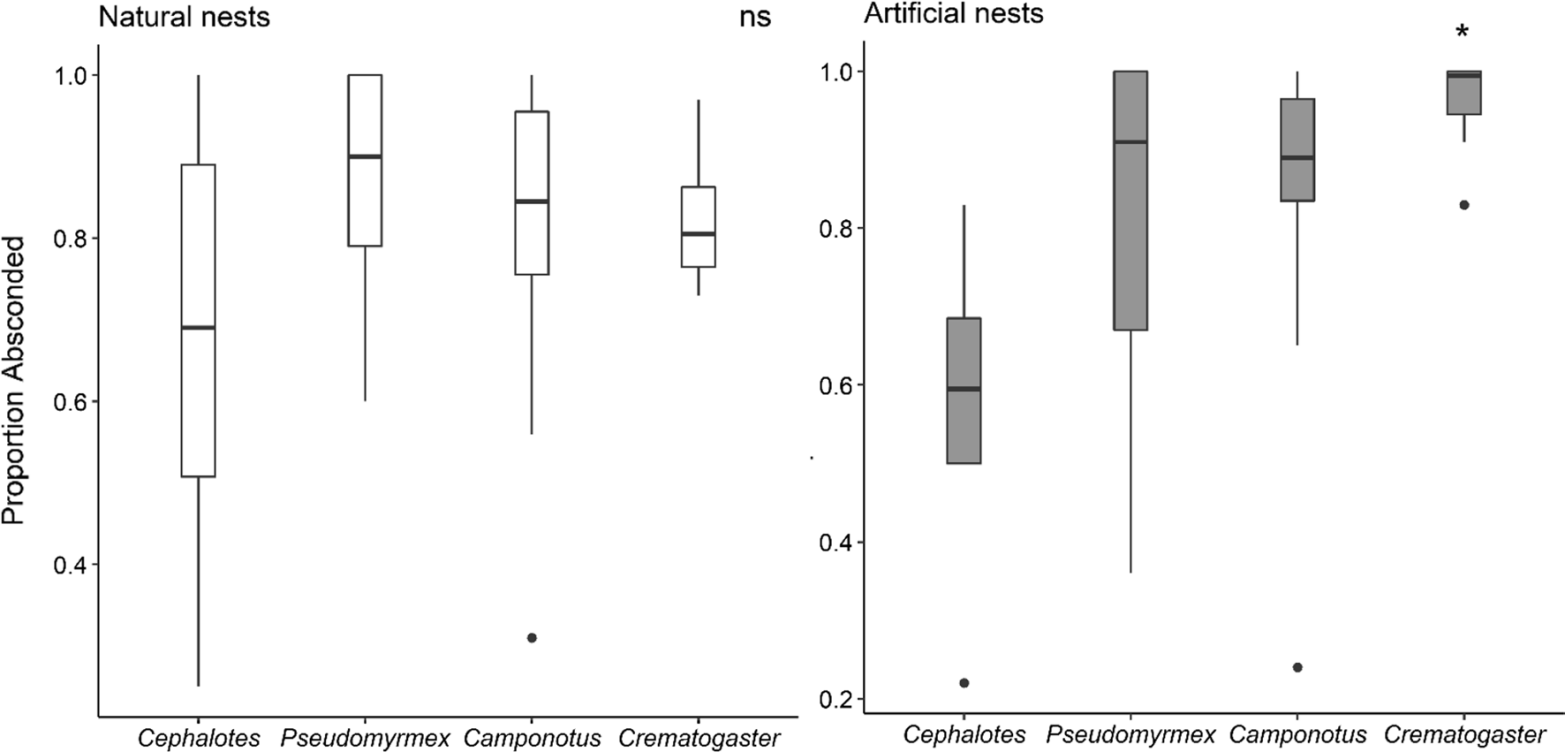
Proportion of the workers that absconded from heated natural and artificial nests. *Crematogaster* absconded from artificial nests in higher proportion than the rest of the genera (*p* < 0.001)

Generally, there was no difference among genera in the proportion of absconded workers in natural nests (Fig. 4), although on average lower proportion of *Cephalotes* absconded (GLMM: *β* = − 1, SE = 0.54, *z* (− 1.9), *p* = 0.0629). By contrast, a significantly higher proportion of *Crematogaster* workers absconded from artificial nests (Fig. 4; GLMM: *β* = 2.5, SE = 0.6, *z* (4.4), *p* < 0.001). Overall, *Cephalotes* had the lowest proportion of workers abandoning heated artificial nests (Fig. 4; GLMM: *β* = − 1.5, SE = 0.75, *z* (− 2.0), *p* = 0.045).

*Camponotus* and *Crematogaster* consistently evacuated brood from heated nests (83% and 90% of cases), whereas this rarely occurred in *Pseudomyrmex* and *Cephalotes* (5% and 17%, respectively). The latter genera generally abandoned their brood when exposed to heat stress. Nests of *Pseudomyrmex* and *Cephalotes* are tightly packed with brood and workers, and workers of *Cephalotes* occasionally were trapped between brood, ultimately leading to their death. *Pseudomyrmex* was the only genus that did not show clear absconding behavior, and the genus with the highest proportional brood content across all genera (Fig. S2; GLMM: *β* = 1.2, SE = 0.4, *z* (3.3), *p* = 0.0008).

Ant behaviors during heating trials were relatively consistent. Typically, a single ant left the nest at the beginning of the trial, explored the exterior of the nest and its surroundings, and then either re-entered the nest or stayed outside. On many occasions, this behavior happened repeatedly before additional workers began to evacuate the nest. Upon exiting the nest, workers cleaned their antennae, and sometimes the first and second pair of legs. Once workers absconded the nest, they usually remained outside, frequently gathering below the nest, or in other cooler areas of the plastic container.

## Discussion

Increasing global and regional temperatures are predicted to be most detrimental for tropical ectotherms (Deutsch et al. 2008; Sunday et al. 2014; Diamond and Chick 2018), while deforestation is continuing to cause extreme warming at the local scale (Zeppetello et al. 2020). Given these changing conditions, ectotherms like ants will increasingly depend on behavioral thermoregulation to avoid heat stress, as many are already living at their physiological thermal limits (Sunday et al. 2014). Behavioral avoidance of thermal extremes is well documented in foraging ants (Marsh 1988; Spicer et al. 2017; Stark et al. 2017; Villalta et al. 2020), but the effects of thermal extremes on nest site occupancy in the tropical forest canopy have been unexplored until now. Here, we show that tropical canopy ants abscond their nest in response to heat stress. *Camponotus* and *Crematogaster*, genera commonly nesting across all forest strata used a broader variety of nesting sites (Fig. 1). *Camponotus* nests tended to heat up faster, which might be why this genus was more likely to evacuate with brood. Heat avoidance strategies differed between genera, suggesting that they might be differentially impacted by heat stress challenges.

Deforestation creates thermally stressful habitats for ants (Boyle et al. 2020) and reduces the number of potential nests sites. This might be particularly damaging for *Cephalotes* and *Pseudomyrmex* which are more selective in their choice of nest sites, and preferentially nest in the canopy. We found that *Cephalotes* nested in smaller cavities that were slower to heat up, suggesting that the choice of nests might mitigate the effect of heat stress. One-way social insects passively thermoregulate their nests by choosing an adequate nest location (Jones and Oldroyd 2006), and this behavior even occurs in army ants, which are nomadic and endothermic (Soare et al. 2011; Baudier et al. 2019). Nest site selection in *Cephalotes* is also governed by the ability to defend their nests (Powell 2016), and *Cephalotes* are able to survive longer in nests that are easier to defend (Powell et al. 2017). Thus, these two nest characteristics—thermoregulatory properties and defensibility—presumably promote nest site specialization.

Absconding temperatures were relatively consistent among three of the focal genera, whereas *Crematogaster* absconded at lower temperatures with a higher proportion of absconded workers. Lower absconding temperature in *Crematogaster* is not caused by lower heat tolerance, as the average critical thermal maximum of *Crematogaster spp.* in this forest is 50 °C (Bujan et al. 2016). A similar pattern was recorded for seed harvesting ant populations, where worker heat tolerance was not a good predictor of behavioral thermoregulation at the colony level (Villalta et al. 2020). Instead, heat stress likely is perceived as disturbance; we consistently observed *Crematogaster* exhibiting gaster flagging defense behavior during heating trials. Given that *Crematogaster* and *Camponotus* brood are a common prey of army ants (Powell and Franks 2006; Hoenle et al. 2019) and absconding is a typical behavioral response to army ant attack, we expected both genera to be especially sensitive to disturbance and thus abscond at lower temperatures. Additionally, brood of these genera might be more sensitive to heat, as brood developmental temperature is lower in ants with lower CT_max_ (Penick et al. 2017).

*Camponotus* workers consistently absconded at 44 °C in both natural and artificial nests. We hypothesize that low variability in absconding temperature in this genus could be due to similar brood rearing requirements in *Camponotus* species, causing their thermoreceptors to be tuned into the same stressful temperature. This merits further examination by testing brood rearing preferences and absconding temperature for each studied species. Moreover, examining relatedness between species could help understanding if these species specific requirements like optimal brood rearing temperature and absconding temperature are phylogenetically constrained.

Differences in worker size contribute to differences in absconding temperature among *Camponotus* and *Crematogaster*. For example, the average *Camponotus* worker is ca. 16 times larger than the average *Crematogaster* worker (Table S2), and thus will heat up more slowly. *Crematogaster* is the smallest of the four tested genera, and among tropical ants, smaller species are generally more prone to heat stress (Kaspari et al. 2015; Baudier et al. 2018). Body size alone may be most important when heating is conductive, as is the case inside a nest, while body color and pilosity together with convective heating impact ant heating rates outside the nest (Spicer et al. 2017). Worker body size did not predict cavity size selection, as both *Camponotus* and *Crematogaster* nested in larger cavities. In contrast, worker size was positively correlated with entrance sizes in cavity-nesting ants that use wood-boring beetle cavities (Priest et al. 2021), potentially because of different nests types and species sampled.

Inside the nest workers are fine-tuned to temperature requirements of the brood and will choose the optimal temperature for brood development (Roces and Núñez 1989). Thermal sensitivity of juvenile insects often differs from the adult stages that are predominantly used in thermal experiments, but data on thermal sensitivity of insect early developmental stages are scarce (Kingsolver and Buckley 2020), ants being no exception (Roeder et al. 2021). Brood is the most thermally sensitive part of an ant colony, and subtropical ants tend to choose a single thermal optimum for brood development (Roces 1995). Mound-building ants actively relocate brood to track changes in nest temperature (Porter and Tschinkel 1993; Penick and Tschinkel 2008; McCaffrey and Galen 2011), but twig-nesting ants have a limited amount of space for brood translocation. However, single colony of canopy ants often occupies multiple nest sites (Powell 2009; Jiménez-Soto and Philpott 2015; Mathis et al. 2016) and such polydomous strategy potentially circumvents this problem. Polydomy was proposed to be one of the mechanisms to avoid thermal stress in ground-nesting *Myrmica*, but with limited support (Banschbach et al. 1997). Generally, polydomy is considered to evolve to maximize resource acquisition or spread the risk from predation, parasitism, or environmental stochasticity (Debout et al. 2007; Robinson 2014). In an already thermally variable habitat, such as the canopy, heat stress might be another mechanism for the evolution of polydomy. Currently, we do not know the optimal brood development temperatures of canopy ants, or how canopy ants choose the number and distribution of satellite nests.

The results of this study show high variability among ant response to experimental heating among congenerics, even across different parts of the same nest. Much of this variation could be a result of differences in the age and caste structure of nest occupants. Manipulation of the number of brood or workers in a nest before heating was not feasible in this study, but would be a useful extension of this project. Likewise, additional data and experiments are needed to determine how often canopy ants experience thermal extremes under current natural conditions, and the role of wood density and other nest characteristics as determinants of nest site selection in canopy ants.

Here, we provide the first experimental examination of behavioral responses to heat stress by tropical canopy ants at the colony level. Considering the importance of ants in ecosystem-level processes, the amount of ant biomass residing in tropical canopies, and the fact that the canopy is the most exposed part of the forest, resolving the behavioral and physiological challenges faced by canopy ants is increasingly important in the face of climatic change and deforestation.

## Supplementary Information

Below is the link to the electronic supplementary material.

Supplementary file1 (DOCX 414 KB)

## Data Availability

Data used in this study are available in Figshare 10.6084/m9.figshare.19298609.v1.

## References

[CR1] Abril S, Oliveras J, Gómez C (2010). Effect of temperature on the development and survival of the argentine ant, *Linepithema humile*. J Insect Sci.

[CR2] Angilletta MJJ (2009). Thermal adaptation: a theoretical and empirical synthesis.

[CR69] Banschbach VS, Levit N, Herbers JM (1997). Nest temperatures and thermal preferences of a forest ant species: is seasonal polydomy a thermoregulatory mechanism?. Insectes Soc.

[CR3] Baudier KM, D’Amelio CL, Malhotra R (2018). Extreme insolation: climatic variation shapes the evolution of thermal tolerance at multiple scales. Am Nat.

[CR4] Baudier KM, D’Amelio CL, Sulger E (2019). Plastic collective endothermy in a complex animal society (army ant bivouacs: *Eciton burchellii parvispinum*). Ecography (cop).

[CR5] Blüthgen N, Stork N (2007). Ant mosaics in a tropical rainforest in Australia and elsewhere: a critical review. Austral Ecol.

[CR6] Boyle MJW, Bishop TR, Luke SH (2020). Localised climate change defines ant communities in human-modified tropical landscapes. Funct Ecol.

[CR7] Bujan J, Yanoviak SP, Kaspari M (2016). Desiccation resistance in tropical insects: causes and mechanisms underlying variability in a Panama ant community. Ecol Evol.

[CR8] Byrne MM (1994). Ecology of twig-dwelling ants in a wet lowland tropical forest. Biotropica.

[CR9] Camarota F, Powell S, Melo AS (2016). Co-occurrence patterns in a diverse arboreal ant community are explained more by competition than habitat requirements. Ecol Evol.

[CR10] Carroll C (1979). A comparative study of two ant faunas : the stem-nesting ant communities of Liberia, West Africa and Costa Rica, Central America. Am Nat.

[CR11] Chick LD, Perez A, Diamond SE (2017). Social dimensions of physiological responses to global climate change: what we can learn from ants (Hymenoptera: Formicidae). Myrmecol News.

[CR12] Clay NA, Lucas J, Kaspari M, Kay AD (2013) Manna from heaven: Refuse from an arboreal ant links aboveground and belowground processes in a lowland tropical forest. Ecosphere 4:art141. 10.1890/ES13-00220.1

[CR13] Davidson D (1997). The role of resource imbalances in the evolutionary ecology of tropical arboreal ants. Biol J Linn Soc.

[CR14] Debout G, Schatz B, Elias M, Mckey D (2007). Polydomy in ants: what we know, what we think we know, and what remains to be done. Biol J Linn Soc.

[CR15] Dejean A, Corbara B, Orivel J, Leponce M (2007). Rainforest canopy ants: the implications of territoriality and predatory behavior. Funct Ecosyst Communities.

[CR16] Dejean A, Corbara B, Roux O, Orivel J (2014). The antipredatory behaviours of neotropical ants towards army ant raids (Hymenoptera: Formicidae). Myrmecol News.

[CR17] Deutsch CA, Tewksbury JJ, Huey RB (2008). Impacts of climate warming on terrestrial ectotherms across latitude. Proc Natl Acad Sci.

[CR18] Diamond SE, Chick LD (2018). Thermal specialist ant species have restricted, equatorial geographic ranges: implications for climate change vulnerability and risk of extinction. Ecography (cop).

[CR19] Dornhaus A, Franks NR, Hawkins RM, Shere HNS (2004). Ants move to improve: colonies of *Leptothorax albipennis* emigrate whenever they find a superior nest site. Anim Behav.

[CR20] Droual R (1983). The Organization of Nest Evacuation in *Pheidole desertorum* Wheeler and *P. hyatti* Emery (Hymenoptera: Formicidae). Behav Ecol Sociobiol.

[CR21] Folgarait P (1998). Ant biodiversity and its relationship to ecosystem functioning: a review. Biodivers Conserv.

[CR22] Ghalambor CK, Huey RB, Martin PR (2006). Are mountain passes higher in the tropics? Janzen’s hypothesis revisited. Integr Comp Biol.

[CR23] Heinrich B (1993). The hot-blooded insects.

[CR24] Hoenle PO, Blüthgen N, Brückner A (2019). Species—level predation network uncovers high prey specificity in a Neotropical army ant community. Mol Ecol.

[CR25] Holm S (1979). A simple sequentially rejective multiple test procedure. Scand J Stat.

[CR26] Horstmann K, Schmid H (1986). Temperature Regulation in Nests of the Wood Ant, *Formica polyctena* (Hymenoptera: Formicidae). Entomol Gen.

[CR27] Janzen D (1967). Why mountain passes are higher in the tropics. Am Nat.

[CR28] Jiménez-Soto E, Philpott SM (2015). Size matters: nest colonization patterns for twig-nesting ants. Ecol Evol.

[CR29] Jones JC, Oldroyd BP (2006) Nest Thermoregulation in social insects. In: Advances in insect physiology. pp 153–191

[CR30] Kaspari M (1996). Litter ant patchiness at the 1–m2 scale: disturbance dynamics in three Neotropical forests. Oecologia.

[CR31] Kaspari M, Powell S, Lattke J, O’Donnell S (2011). Predation and patchiness in the tropical litter: do swarm-raiding army ants skim the cream or drain the bottle?. J Anim Ecol.

[CR32] Kaspari M, Clay NA, Lucas J (2015). Thermal adaptation generates a diversity of thermal limits in a rainforest ant community. Glob Chang Biol.

[CR33] Kingsolver JG, Buckley LB (2020). Ontogenetic variation in thermal sensitivity shapes insect ecological responses to climate change. Curr Opin Insect Sci.

[CR34] Kumagai T, Kuraji K, Noguchi H (2001). Vertical profiles of environmental factors within tropical rainforest, Lambir Hills National Park, Sarawak, Malaysia. J for Res.

[CR35] Kuznetsova A, Brockhoff PB, Christensen RHB (2017). lmerTest package: tests in linear mixed effects models. J Stat Softw.

[CR36] Le Breton J, Dejean A, Snelling G, Orivel J (2007). Specialized predation on *Wasmannia auropunctata* by the army ant species *Neivamyrmex compressinodis*. J Appl Entomol.

[CR37] Leigh EGJ (1999) Tropical Forest Ecology: A View from Barro Colorado Island

[CR38] Lenth R, Singmann H, Love J et al (2018) Package ‘emmeans’. Estimated marginal measn, aka least-squares means. R Packag. version 1.15–15

[CR39] Marsh AC (1988). Activity patterns of some Namib Desert ants. YJARE.

[CR40] Mathis KA, Philpott SM, Ramirez SR (2016). Variation in spatial scale of competing polydomous twig-nesting ants in coffee agroecosystems. Insectes Soc.

[CR41] McCaffrey J, Galen C (2011). Between a rock and a hard place: impact of nest selection behavior on the altitudinal range of an alpine ant, *Formica neorufibarbis*. Environ Entomol.

[CR42] Oms CS, Cerdá X, Boulay R (2017). Is phenotypic plasticity a key mechanism for responding to thermal stress in ants?. Sci Nat.

[CR43] Penick CA, Tschinkel WR (2008). Thermoregulatory brood transport in the fire ant, *Solenopsis invicta*. Insectes Soc.

[CR44] Penick CA, Diamond SE, Sanders NJ, Dunn RR (2017). Beyond thermal limits: comprehensive metrics of performance identify key axes of thermal adaptation in ants. Funct Ecol.

[CR45] Philpott S, Armbrecht I (2006). Biodiversity in tropical agroforests and the ecological role of ants and ant diversity in predatory function. Ecol Entomol.

[CR46] Philpott S, Foster P (2005). Nest-site limitation in coffee agroecosystems: artificial nests maintain diversity of arboreal ants. Ecol Appl.

[CR47] Porter SD, Tschinkel WR (1993). Fire ant thermal preferences: behavioral control of growth and metabolism. Behav Ecol Sociobiol.

[CR48] Powell S (2009). How ecology shapes caste evolution: linking resource use, morphology, performance and fitness in a superorganism. J Evol Biol.

[CR49] Powell S (2016). A comparative perspective on the ecology of morphological diversification in complex societies: nesting ecology and soldier evolution in the turtle ants. Behav Ecol Sociobiol.

[CR50] Powell S, Franks NR (2006). Ecology and the evolution of worker morphological diversity: a comparative analysis with *Eciton* army ants. Funct Ecol.

[CR51] Powell S, Donaldson-Matasci M, Woodrow-Tomizuka A, Dornhaus A (2017). Context-dependent defences in turtle ants: resource defensibility and threat level induce dynamic shifts in soldier deployment. Funct Ecol.

[CR52] Priest GV, Camarota F, Powell S (2021). Ecosystem engineering in the arboreal realm: heterogeneity of wood—boring beetle cavities and their use by cavity-nesting ants. Oecologia.

[CR53] R Core Team (2019) R: a language and environment for statistical computing. Vienna, Austria

[CR54] Robinson EJH (2014). Polydomy: the organisation and adaptive function of complex nest systems in ants. Curr Opin Insect Sci.

[CR55] Roces F (1995). Variable thermal sensitivity as output of a circadian clock controlling the bimodal rhythm of temperature choice in the ant *Camponotus mus*. J Comp Physiol A.

[CR56] Roces F, Núñez JA (1989). Brood translocation and circadian variation of temperature preference in the ant *Camponotus mus*. Oecologia.

[CR57] Roeder KA, Roeder DV, Bujan J (2021). Ant thermal tolerance: a review of methods, hypotheses, and sources of variation. Ann Entomol Soc Am.

[CR58] Soare TW, Tully SI, Willson SK (2011). Choice of nest site protects army ant colonies from environmental extremes in tropical montane forest. Insectes Soc.

[CR59] Spicer ME, Stark AY, Adams BJ (2017). Thermal constraints on foraging of tropical canopy ants. Oecologia.

[CR60] Stark AY, Adams BJ, Fredley JL, Yanoviak SP (2017). Out on a limb: thermal microenvironments in the tropical forest canopy and their relevance to ants. J Therm Biol.

[CR61] Sunday JM, Bates AE, Kearney MR (2014). Thermal-safety margins and the necessity of thermoregulatory behavior across latitude and elevation. Proc Natl Acad Sci.

[CR62] Tanaka HO, Yamane S, Itioka T (2010). Within-tree distribution of nest sites and foraging areas of ants on canopy trees in a tropical rainforest in Borneo. Popul Ecol.

[CR63] Tschinkel WR (1987). Seasonal life history and nest architecture of a winter-active ant, *Prenolepis imparis*. Insectes Soc.

[CR64] Villalta I, Oms CS, Angulo E (2020). Does social thermal regulation constrain individual thermal tolerance in an ant species?. J Anim Ecol.

[CR65] Warren R, Price J, Graham E (2018). The projected effect on insects, vertebrates, and plants of limiting global warming to 1.5°C rather than 2°C. Science (80-).

[CR66] Winston ML, Otis GW, Taylor OR (1979). Absconding behaviour of the africanized honeybee in south america. J Apic Res.

[CR67] Wright SJ (2005). Tropical forests in a changing environment. Trends Ecol Evol.

[CR68] Zeppetello LRV, Parsons LA, Spector JT (2020). Large scale tropical deforestation drives extreme warming. Environ Res Lett Lett.

