# Supplementary material for: Behavioral response to heat stress of twig-nesting canopy ants
Source: Oecologia. 2022 Mar 7;198(4):947–55. doi: 10.1007/s00442-022-05143-6 (PMC9056446; doi:10.1007/s00442-022-05143-6)
Supplement: Supplementary file 1 — Supplementary file1 (DOCX 414 KB) [file 442_2022_5143_MOESM1_ESM.docx]

# **Supplementary information to:**

Bujan J* & Yanoviak SP. *Behavioral response to heat stress of twig nesting canopy ants*

*corresponding author: jelena.bujan@unil.ch

Table S1. List of species tested for each genus in trials with natural nests (A) and artificial nests (B). Number of nests tested (N) is provided for each species and genus. Nest site includes nesting locations we recorded in our sampling. For species that we collected only once nest site is a single recorded location, and might not fully reflect species nesting preference.

| **A) Natural Nests** | | |  |  | |
| --- | --- | --- | --- | --- | --- |
| **GENUS** | **SPECIES** | **N** | **NEST SITE** | |  |
| ***Camponotus* (N=24)** | brett_LH | 3 | Both |  | |
|  | *brevis* | 4 | Understory |  | |
|  | *claviscapus* | 1 | Canopy |  | |
|  | *excisus* | 1 | Understory |  | |
|  | JTL005 | 1 | Understory |  | |
|  | *mucronatus* | 4 | Both |  | |
|  | *novogranadensis* | 1 | Both |  | |
|  | *platanus* | 2 | Both |  | |
|  | *senex* | 5 | Both |  | |
|  | *sexguttatus* | 1 | Canopy |  | |
|  | sp. 1 | 1 | Understory |  | |
| ***Cephalotes* (N=17)** | *christopherseni* | 11 | Canopy |  | |
|  | *minutus* | 2 | Canopy |  | |
|  | *porrasi* | 2 | Canopy |  | |
|  | *umbraculatus* | 2 | Canopy |  | |
| ***Crematogaster* (N=8)** | *brasiliensis* | 2 | Understory |  | |
|  | *carinata* | 2 | Both |  | |
|  | *erecta* | 1 | Canopy |  | |
|  | *limata* | 3 | Both |  | |
| ***Pseudomyrmex* (N=15)** | shiny_Ybrown_ | 1 | Canopy |  | |
|  | *boopis* | 1 | Understory |  | |
|  | *duckei* | 1 | Canopy |  | |
|  | *elongatus* | 3 | Canopy |  | |
|  | *euryblemma* | 1 | Canopy |  | |
|  | *gracilis* | 5 | Both |  | |
|  | *oculatus* | 2 | Canopy |  | |
|  | *simplex* | 1 | Canopy |  | |

Table S1. (continued)

| **B) Artificial Nests** | | |  |
| --- | --- | --- | --- |
| **GENUS** | **SPECIES** | **N** |  |
| ***Camponotus* (N=22)** | brett_LH | 3 | Both |
|  | *brevis* | 9 | Understory |
|  | *mucronatus* | 5 | Both |
|  | *senex* | 2 | Both |
|  | *sexguttatus* | 1 | Canopy |
|  | sp. 1 | 2 | Canopy |
| ***Cephalotes* (N=8)** | *minutus* | 6 | Canopy |
|  | *porrasi* | 2 | Understory |
| ***Crematogaster* (N=18)** | *brasiliensis* | 5 | Understory |
|  | *carinata* | 8 | Both |
|  | *limata* | 5 | Both |
| ***Pseudomyrmex* (N=16)** | *boopis* | 2 | Understory |
|  | *ejectus* | 1 | Canopy |
|  | *elongatus* | 1 | Canopy |
|  | *gracilis* | 8 | Both |
|  | *ita* | 1 | Canopy |
|  | *oculatus* | 3 | Canopy |

Table S2. Genera tested in the study with their average body size and CT_max_—critical thermal maximum in °C (from Kaspari et al. 2015, Bujan et al. 2016).

| SUBFAMILY | GENUS | BODY SIZE (mg) | CT_max_ |
| --- | --- | --- | --- |
| Formicinae | *Camponotus* | 2.059 | 47 |
| Myrmicinae | *Crematogaster* | 0.125 | 50 |
| Myrmicinae | *Cephalotes* | 3.544 | 49 |
| Pseudomyrmicinae | *Pseudomyrmex* | 1.626 | 52 |

Table S3. Maximum nest temperatures (T_max_) measured in the field for natural nests of different lengths and diameters placed in tree crowns. Temperatures inside of nests exceed 40°C in 12/51 nests.

| Length (cm) | Diamenter (mm) | DATE | TIME | Tmax |
| --- | --- | --- | --- | --- |
| 24 | 3.8 | 26-Jan-19 | 10:36:12 | 42 |
| 17 | 12 | 4-Feb-19 | 13:43:06 | 41 |
| 17 | 8 | 12-Jan-19 | 16:55:45 | 41 |
| 24 | 3.8 | 25-Jan-19 | 10:08:22 | 41 |
| 17 | 8 | 26-Jan-19 | 12:31:42 | 41 |
| 17 | 12 | 26-Jan-19 | 12:31:56 | 40 |
| 17 | 12 | 27-Jan-19 | 15:28:15 | 40 |
| 17 | 8 | 27-Jan-19 | 11:05:15 | 40 |
| 17 | 11 | 30-Jan-19 | 12:29:54 | 40 |
| 17 | 8 | 25-Jan-19 | 12:23:58 | 40 |
| 17 | 12 | 25-Jan-19 | 12:36:34 | 40 |
| 17 | 8 | 13-Jan-19 | 15:54:57 | 40 |
| 17 | 8 | 30-Jan-19 | 10:44:35 | 39 |
| 18 | 6.5 | 30-Jan-19 | 12:29:24 | 39 |
| 17 | 12 | 31-Jan-19 | 14:14:50 | 38 |
| 17 | 12 | 12-Jan-19 | 16:14:37 | 38 |
| 24 | 3.8 | 30-Jan-19 | 10:30:45 | 38 |
| 24 | 3.8 | 4-Feb-19 | 13:33:42 | 38 |
| 18 | 5 | 30-Jan-19 | 13:47:50 | 37 |
| 17 | 12 | 30-Jan-19 | 10:45:05 | 37 |
| 17 | 8 | 29-Jan-19 | 12:39:32 | 37 |
| 18 | 6.5 | 31-Jan-19 | 10:51:32 | 37 |
| 24 | 3.8 | 12-Jan-19 | 15:49:27 | 37 |
| 17 | 11 | 31-Jan-19 | 10:59:26 | 37 |
| 24 | 3.8 | 29-Jan-19 | 10:34:18 | 36 |
| 17 | 12 | 13-Jan-19 | 16:25:19 | 36 |
| 17 | 10 | 30-Jan-19 | 12:29:16 | 36 |
| 15 | 5 | 23-Jan-19 | 12:30:18 | 36 |
| 24 | 3.8 | 13-Jan-19 | 16:08:41 | 35 |
| 17 | 8 | 4-Feb-19 | 13:42:38 | 35 |
| 17 | 12 | 29-Jan-19 | 12:39:38 | 35 |
| 17 | 10 | 31-Jan-19 | 11:17:46 | 34 |
| 15 | 5 | 25-Jan-19 | 12:30:44 | 33 |
| 18 | 5 | 31-Jan-19 | 11:08:08 | 33 |
| 15 | 5 | 28-Jan-19 | 15:14:25 | 33 |
| 24 | 3.8 | 31-Jan-19 | 12:13:54 | 33 |
| 17 | 8 | 31-Jan-19 | 12:14:02 | 33 |
| 15 | 10 | 23-Jan-19 | 11:31:10 | 33 |
| 15 | 10 | 28-Jan-19 | 15:14:25 | 33 |
| 15 | 10 | 25-Jan-19 | 11:44:44 | 32 |
| 15 | 10 | 23-Jan-19 | 9:59:04 | 32 |
| 15 | 10.5 | 28-Jan-19 | 15:14:25 | 32 |
| 15 | 10 | 28-Jan-19 | 15:15:37 | 32 |
| 15 | 10.5 | 23-Jan-19 | 11:11:26 | 32 |
| 15 | 10.5 | 25-Jan-19 | 13:46:06 | 32 |
| 15 | 10 | 25-Jan-19 | 12:10:08 | 30 |
| 17 | 10 | 29-Jan-19 | 14:20:07 | 29 |
| 17 | 11 | 29-Jan-19 | 14:20:21 | 29 |
| 18 | 6.5 | 29-Jan-19 | 14:20:19 | 29 |
| 18 | 5 | 29-Jan-19 | 14:28:33 | 29 |

Figure S1. Experimental setup for nest heating trials. Three thermocouples measured temperature inside the nest (a), on the underside of the nest (b), and nearby air inside the box (c).


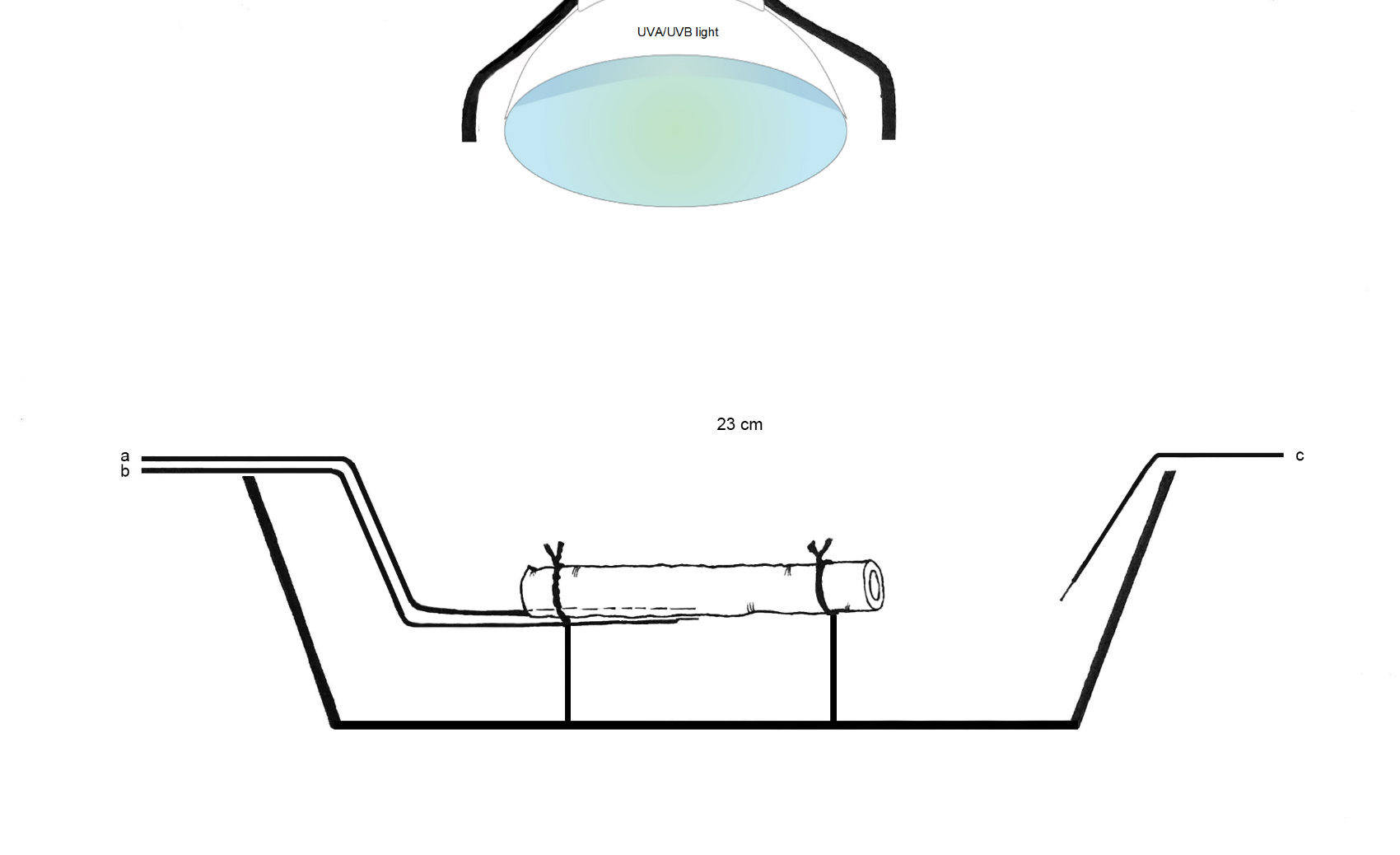


Figure S2. Proportion of brood and the total nest content (brood + workers) across four focal genera in natural and artificial nests combined.


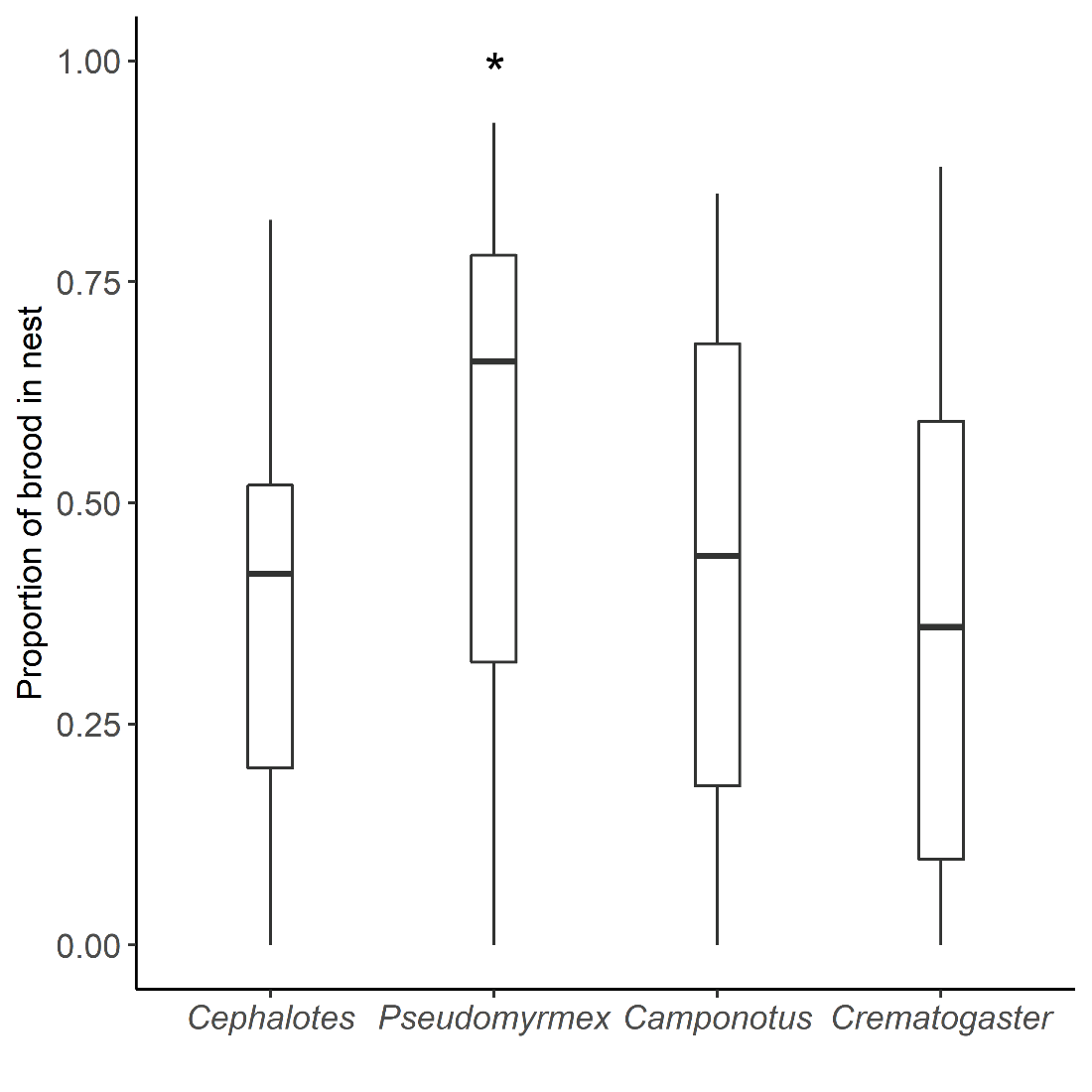


**References:**

Bujan J, Yanoviak SP, Kaspari M (2016) Desiccation resistance in tropical insects: causes and mechanisms underlying variability in a Panama ant community. Ecol Evol 6:6282–6291. https://doi.org/10.1002/ece3.2355

Kaspari M, Clay NA, Lucas J, et al (2015) Thermal adaptation generates a diversity of thermal limits in a rainforest ant community. Glob Chang Biol 21:1092–1102. https://doi.org/10.1111/gcb.12750
